# Treatment-Resistant Depression – What is the Effective Maintenance Treatment

**DOI:** 10.1192/j.eurpsy.2022.1422

**Published:** 2022-09-01

**Authors:** D. Kolar

**Affiliations:** Queen’s University, Department Of Psychiatry, Kingston, Canada

**Keywords:** treatment resistant depression, ECT, rTMS, ketamine

## Abstract

**Introduction:**

Treatment-resistant depression (TRD) presents a significant challenge in clinical practice. Besides antidepressant medications, neurostimulation methods (ECT, rTMS) and ketamine are viable treatment options.

**Objectives:**

To objectively evaluate the real effectiveness of treatments within interventional psychiatry in the maintenance treatment.

**Methods:**

The extensive literature review of the efficacy of ECT, rTMS, and ketamine treatment in the maintenance treatment of TRD and the author’s clinical and research experience will be included in this presentation.

**Results:**

Neurostimulation, particularly ECT and ketamine treatment are usually effective treatments for patients with TRD. However, both of these treatment modalities do not have sustained benefits and after discontinuing treatment the majority of patients relapse. Ketamine has rapid therapeutic effects in depression, but these effects are short-lived. Continuation treatment with ketamine in the form of intranasal ketamine is an option, but concerns over cognitive impairment, interstitial cystitis and significant addictive potential related to longer use of ketamine are significant limiting factors. rTMS is a first-line treatment option for patients with TRD according to the Canadian CANMAT guidelines. However, the majority of patients may relapse following the course of rTMS. The maintenance rTMS over an extended period of time is usually not feasible as it may significantly affect the waiting time for newly referred patients. Portable TMS machine for home use would be an alternative option for a limited number of patients.

**Conclusions:**

Maintenance treatment has been always a big clinical challenge in mood disorder psychiatry. Only well-established multimodal treatment is a realistic option for getting long-term benefits in treating patients with TRD.

**Disclosure:**

No significant relationships.

